# The Impact of Digital Economy on the Economic Growth and the Development Strategies in the post-COVID-19 Era: Evidence From Countries Along the “Belt and Road”

**DOI:** 10.3389/fpubh.2022.856142

**Published:** 2022-05-09

**Authors:** Jinzhu Zhang, Wenqi Zhao, Baodong Cheng, Aixin Li, Yanzhuo Wang, Ning Yang, Yuan Tian

**Affiliations:** ^1^Department of Agricultural and Forestry Economics and Management, School of Economics and Management, Beijing Forestry University, Beijing, China; ^2^Department of International Trade, School of Economics and Management, Beijing Forestry University, Beijing, China; ^3^Department of International Business, Business College, Beijing Union University, Beijing, China; ^4^Beijing Shenzhou Chiji Fund Management Co., Ltd., Beijing, China; ^5^School of Mathematics and Physics, Faculty of Science, The University of Queensland, Brisbane, QLD, Australia

**Keywords:** digital economy, economic growth, trade pattern, COVID-19, countries along the “Belt and Road”

## Abstract

The digital economy is considered as an effective measure to mitigate the negative economic impact of the Corona Virus Disease 2019 (COVID-19) epidemic. However, few studies evaluated the role of digital economy on the economic growth of countries along the “Belt and Road” and the impact of COVID-19 on their digital industries. This study constructed a comprehensive evaluation index system and applied a panel data regression model to empirically analyze the impact of digital economy on the economic growth of countries along the “Belt and Road” before COVID-19. Then, a Global Trade Analysis Project (GTAP) model was used to examine the impact of COVID-19 on their digital industries and trade pattern. Our results show that although there is an obvious regional imbalance in the digital economy development in countries along the “Belt and Road”, the digital economy has a significantly positive effect on their economic growth. The main impact mechanism is through promoting industrial structure upgrading, the total employment and restructuring of employment. Furthermore, COVID-19 has generally boosted the demand for the digital industries, and the impact from the demand side is much larger than that from the supply side. Specifically, the digital industries in Armenia, Israel, Latvia and Estonia have shown great growth potential during the epidemic. On the contrast, COVID-19 has brought adverse impacts to the digital industries in Ukraine, Egypt, Turkey, and the Philippines. The development strategies are proposed to bridge the “digital divide” of countries along the “Belt and Road,” and to strengthen the driving effect of the digital economy on industrial upgrading, employment and trade in the post-COVID-19 era.

## Introduction

Digital technologies, typically represented by the Internet, big data, 5G, artificial intelligence, accelerate the deep integration with industries, bringing the world into the era of digital economy (1). Since the “Belt and Road” initiative was proposed, the digital economy has also gradually become a crucial cooperation area for the countries (2). During the 4th World Internet Conference in 2017, the “Belt and Road” Digital Economy International Cooperation Initiative was launched, which aimed to build an interconnected “digital silk road” and to create a “community of interests and destiny” (3). The digital economy can further optimize the industrial structure and increase jobs through information and communications technologies (ICT), Internet and other intelligent means, greatly improving the economic development in countries along the “Belt and Road.” In particular, the digital economy has played an active role in mitigating economic losses and promoting economic recovery during the fight against Corona Virus Disease 2019 (COVID-19). Specifically, COVID-19 brought serious shocks to the world economy by directly affecting production, disrupting the supply chain and having an adverse impact on firms and financial markets (4–7). Additionally, the stringency measures implemented by policymakers to minimize social mobility also decrease macroeconomic activity (8). On the contrast, the digital economy, with its advantages of high technology and integration with other industries, has become a new opportunity for digital transformation of industries. Compared with the real economy, digital technologies, industries and services play an important role as stabilizers, lubricants and boosters (9). Thus, they are considered as important measures to combat the crisis and engines to drive economic growth. However, the digital economy development in countries along the “Belt and Road” still varies greatly, resulting in their inequitable access to digital development opportunities. Therefore, with the trend of digital transformation in the post-COVID-19 era, it is necessary to assess the digital economy development in countries along the “Belt and Road,” reveal its impact mechanism on economic growth and clarify the impact of COVID-19 on digital economy-related industries. This can provide a policy reference for further strengthening the digital economy cooperation of countries along the “Belt and Road” in the post-COVID-19 era and narrowing the “digital divide” with developed countries.

Considering that the global economic governance is entering the post-COVID-19 era along with the digital transformation, this paper attempts to study the mechanism of the impact of digital economy on economic development and explores the development strategies in the post-COVID-19 era. With the rise of emerging technologies such as big data, cloud computing, and the Internet of Things, ICT is gradually considered as the “engine” for economic development (10–12). However, from the existing studies, there is no consistent conclusion about the impact of the digital economy on the national economy. Some scholars argued that the development of the digital economy could improve the efficiency of factors such as capital and labor, thus contributing to economic growth (13, 14). In addition, the digital economy, as an emerging development model, represents a change in the way of economic growth, which will have a positive impact on the employment and industrial structure, thus affecting the economic development (14). However, other scholars argued that the cost of ICT development and use is expensive due to the lack of infrastructure, especially for less developed countries (15). Therefore, there is a wide divergence of conclusions related to the digital economy on economic development, and research on the impact mechanism of the digital economy on economic development is very limited. After the outbreak of COVID-19, the role of the digital economy on economic recovery has further attracted the attention of scholars. It has been documented that COVID-19 prompted a rapid shift of consumer demand online, creating opportunities for emerging digital industries (16, 17). These online services can reduce the movement of people, reduce the risk of epidemic transmission, and also contribute to stable economic growth. However, current research is still dominated by qualitative analysis, and quantitative assessment of the impact of COVID-19 on the digital economy is less available.

Based on the existing literature, we find that the relationship between the digital economy and the economic development remains ambiguous, and the impact mechanism needs to be further investigated. In addition, few studies have assessed how much COVID-19 has impacted the digital economy across countries along the “Belt and Road.” Therefore, it is not clear how the digital economy of these countries should develop in the future. To this end, this paper made the following contributions to address the current research gap: First, this paper establishes a comprehensive index system to reflect the differences in digital economy infrastructure, openness, innovation environment and competitiveness among countries along the “Belt and Road.” Second, a panel data regression model and a mediating effects model are used to reveal the impact mechanism of the digital economy on the economic growth through the adjustment of industrial structure and improvement of employment before COVID-19. This can provide historical experience and evidence for the use of the digital economy to promote high-quality economic growth in countries along the “Belt and Road.” Finally, the Global Trade Analysis Project (GTAP) model is used to examine the opportunities or challenges brought by the supply-side and demand-side impacts of COVID-19 to the digital industries. This will provide policy insights to better utilize the digital economy development opportunities to mitigate economic losses and promote the transformation of digital industries in the post-COVID-19 era.

The remainder of this paper is organized as follows. Section Literature Review conducts a review of literature related to the impact of digital economy. Section Measurement of Digital Economy Development measures the digital economy development in countries along the “Belt and Road.” Section Digital Economy's Impact Before COVID-19 examines the mechanism of the impact of the digital economy on the economic growth of countries along the “Belt and Road” before COVID-19. Section COVID-19's impact on digital economy discusses the impact of COVID-19 on the digital economy and trade patterns. The final section is Conclusions and Policy Implications.

## Literature Review

In recent years, the digital economy has become a new economic form after the agricultural and industrial economies (14). The concept of the digital economy was first proposed by Tapscott (18), who indicated that the age of networked intelligence is not only about the networking of technology, but about the networking of humans through technology. The integration of digital and network technologies has made the digital economy prominent in economic and social activities; thus its connotation has become richer. Mesenbourg (19) defined the digital economy in terms of three components: e-business infrastructure, e-business and e-commerce. Other scholars considered the digital economy as a dynamic process instead of static efficiency (20). In recent years, the digital economy was defined as a wider than modest digitizing segment, and its general meanings integrate all the digitally-oriented economic activities (21, 22). For instance, the Organization for Economic Co-operation and Development (OECD) described the concept of the digital economy as “the digital transformation of economic and social development” and considered all traditional industries in the process of digitization and networking as part of the digital economy (23). The G20 Digital Economy Development and Cooperation Initiative further defined the digital economy as “a broad range of economic activities that include using digitized information and knowledge as the key factor of production, modern information networks as an important activity space, and the effective use of ICT as an important driver of productivity growth and economic structural optimization” (24). Therefore, the ambiguous definition of digital economy leads to its inconsistent measurement index system.

Previous studies have shown that the digital economy is considered the main driver of economic growth in both developed and developing countries (25, 26). The digital economy mainly based on ICT helps to increase capital and labor productivity and to obtain goods and services at lower prices (13). For example, Seo et al. (27) developed a cumulative growth model to examine the positive relationship between ICT investment and economic growth in 29 countries and found that countries with relatively low levels of productivity could take advantage of the knowledge spillover effects of ICT to close the gap with developed countries. Vu (10) also found that ICT can increase the output by facilitating technology innovation, improving the quality of decision-making, and reducing production costs. With the rapid development of digital technologies such as ICT, more and more scholars have focused on the role of the digital economy on consumer surplus (28), e-commerce supply chain (29), and smart cities (30). Especially after the outbreak of COVID-19, the role of the digital economy on economic recovery has attracted the attention of scholars. Some scholars suggested that the digital economy played a hugely positive role in pandemic prevention and control, value-added distribution in global value chains, and economic development (31). During the COVID-19 pandemic, digital services received a large portion of the resources reallocated from traditional industries, which became a strong driver for accelerated growth (32). In addition, Jiang (33) found that digital technologies not only empowered pandemic response strategies in the short term but also served as the technological foundation for Internet-based industry and consumption in the long term. However, other scholars have suggested that the digital economy may be detrimental to economic growth, especially in the absence of economic transition (34, 35). Although COVID-19 served as an accelerator in advancing the adoption of various technologies, this process had been contested and the outcomes remained uncertain (36).

It can be seen that in the post-COVID-19 era, developing the digital economy can be both a “booster” for the regional economy and a threat to other sectors. Based on the existing literature, this paper identified some research gaps in the current literature. First, the definition of the digital economy has not yet been reached a consensus, and its index system is inadequate. While the existing literature focuses on analyzing the impact of ICT on economic development in terms of the number of Internet users, fixed broadband Internet users, and mobile subscribers. These indicators cannot fully reflect the broader connotations of the digital economy. Moreover, studies have mostly explored the role of digitalization on economic development and provided ambiguous conclusions. However, few studies have focused on the impact mechanism of the digital economy on the economic growth of the countries along the “Belt and Road.” The digital economy is gradually becoming an important area of cooperation for countries along the “Belt and Road.” Analyzing the impact mechanism of the digital economy on their economic growth can provide a reference for the economic recovery and growth in the post-COVID-19 era. More importantly, although some scholars have realized that the epidemic has brought new opportunities and challenges to the digital economy, fewer studies have quantitatively assessed the impact of COVID-19 on the digital economy of countries along the “Belt and Road.”

## Measurement of Digital Economy Development

In order to assess the impact of digital economy development on the economic growth of countries along the “Belt and Road” before COVID-19, this paper needs to measure the digital economy development of these countries. First, we build a comprehensive evaluation index system based on the concept and characteristics of digital economy from three dimensions: digital economy infrastructure, digital economy openness, and innovation environment and competitiveness required for digital technology development. Then the factor analysis and principal component analysis are used to calculate the weights and comprehensive scores of digital economy indicators from 2009 to 2019. Furthermore, the differences in digital economy development in countries along the “Belt and Road” before COVID-19 are analyzed.

This study refers to Belt and Road Portal (https://www.yidaiyilu.gov.cn/index.htm), and divides the countries along the “Belt and Road” into seven plates according to their geographical locations, including China, 5 countries in Central Asia, 2 countries in North Asia, 8 countries in South Asia, 11 countries in Southeast Asia, 19 countries in Central and Eastern Europe, and 19 countries in West Asia and the Middle East (see Appendix Table 1). This study selects 31 countries along the “Belt and Road,” owning to the data availability and sample representativeness.

### Selection of Evaluation Indicators

Before establishing the digital economy development index system, we need to define the concept of digital economy. According to the existing literature, the digital economy in the narrow scope refers to the information technology (IT) or ICT sector producing foundational digital goods and services (37), and the digital economy in a broad scope integrates all the digitally oriented economic activities, which takes the digitization of ICT as a pivotal production factor, uses modern information and communication infrastructure as a carrier, and provides products or services with digital technologies (21, 38). Therefore, the index system of digital economy development is constructed on the broad concept and the necessary conditions required for its development. Referring to the studies of Ershova et al. (39), Kuzovkova et al. (40), Ashmarina et al. (41), Szeles and Simionescu (42) and considering the availability of data, this paper selects indicators from three dimensions: digital economy infrastructure, digital economy openness, and digital technology innovation environment and competitiveness. This index system can reflect the development of ICT technology and industry, as well as the factor endowment conditions and innovation environment required for the development of digital economy. Among them, digital economy infrastructure mainly reflects the foundation and applications of digital technology; innovation environment and competitiveness reflect the R&D capacity and environment in the field of digital economy; digital economy openness reflects the international competitiveness of products produced by digital industry. Each dimension is composed of specific indicators (see Table 1).

**Table 1:** Comprehensive evaluation index system of digital economic development.

**Categories**	**Name of indicators**	**Meaning of indicators**	**The scale value**	**Data sources**
Digital economy infrastructure	Secure Internet servers (per million people)	Network environment security, the government network supervision and governance	0.3–122481.4	World Bank Database
	Fixed broadband subscriptions (per 100 people)	Improvement of the information infrastructure	0.2–39.3	World Bank Database
	Fixed telephone subscriptions (per 100 people)	Improvement of the information infrastructure	1.2–54.8	World Bank Database
	Mobile cellular subscriptions (per 100 people)	Improvement of the information infrastructure	43.1–191.1	World Bank Database
	Individuals using the Internet (percentage of population)	Internet user base	5.1–95.8	World Bank Database
Digital economy openness	High-tech exports (percentage of the manufactured goods exports)	Openness of the digital economy, international competitiveness of technology	0.5–53.3	World Bank Database
	ICT product exports (percentage of total product exports)	Openness of the digital economy, international competitiveness of technology	0–36.5	World Bank Database
Digital technology innovation environment and competitiveness	Enrollment in higher education institutions (percentage of total population)	Abundance of the digital professionals	6.7–148.9	World Bank Database
	R&D (research and development) expenditures (percentage of GDP)	Digital technology innovation environment	0–5.1	World Bank Database
	Availability of the latest technologies	Technology transformation and effective utilization	3.4–6.5	World Bank Database
	Venture capital availability	Suitability of the innovation environment	1.6–5.2	Global Competitiveness Report

(1) The first category of indicators mainly reflects a country's digital economy infrastructure and applications, including secure Internet servers (per million people) (X2), fixed broadband subscriptions (per 100 people) (X7), fixed telephone subscriptions (per 100 people) (X8), Mobile cellular subscriptions (per 100 people) (X10), and Individuals using the Internet (percentage of population) (X9).

(2) The second category of indicators measures the development of a country's ICT industry and its international market share, and also reflects the degree of outward orientation of the digital economy. It includes two indicators: high-tech exports (percentage of the manufactured goods exports) (X3) and ICT product exports (percentage of total product exports) (X4).

(3) The third category of indicators reflects the innovation environment and competitiveness of a country's digital technology, including enrollment in higher education institutions (percentage of total population) (X1), R&D expenditures (percentage of GDP) (X5), venture capital availability(X11), and availability of the latest technologies (X6). The production factors in the digital economy are not only capital and labor, but R&D investment embodied in digital information and specialized technical talents who master digital knowledge. Therefore, this paper considers higher education enrollment and R&D expenditures as factors reflecting the human capital and innovation competitiveness required for the development of the digital economy. Venture capital availability and latest technology availability reflect the suitability for innovation and the transformation of new technological achievements. The higher the availability of venture capital, the more conducive to the innovative activities in the digital economy. Availability of latest technologies reflects a country's innovation transformation rate and the business environment. Without a favorable business environment, it is not necessary to expect “digital dividends” and realize all the opportunities offered by digital technologies.

### Calculation Results of Digital Economy

We use the index system in Section Selection of evaluation indicators to measure the digital economy development of countries along the “Belt and Road.” Each indicator value is multiplied by the corresponding weight and summed up to obtain a country's comprehensive score of digital economy. First, the data related to digital economy indicators are standardized, then Pearson correlation test, Kaiser-Meyer-Olkin (KMO) and Bartlett's sphericity test are performed to determine whether the data are suitable for factor analysis. Second, factor analysis is performed on the data of each indicator using the maximum variance rotation method to extract the top three principal components whose cumulative contribution of variance exceeded 70%. Finally, the factor loading matrix is calculated using principal component analysis to obtain the scores of the three principal factors. The variance contribution rate corresponding to each principal factor is used as the weight to obtain the comprehensive digital economy score of each country. Countries with scores of 70 and above are considered to have a high level of digital economy development; scores of 30–70 are at a medium level; scores below 30 indicate that the development of digital economy is lagging behind.

#### Indicator Correlation Test

The digital economy-related indicators must be standardized and given reasonable weights, and then the selected indicators are multiplied by the corresponding weights and added up, through which the digital economic development scores of countries along the “Belt and Road” can be calculated. This study uses factor analysis to determine the intrinsic correlations and weights of 11 indicators and applies SPSS22.0 software to conduct a Pearson correlation test, as well as KMO and Bartlett's sphericity test on the 11 indicators to determine whether the data selected in this study is suitable for factor analysis. According to the results of the Pearson correlation test, the 11 selected indicators are significantly correlated, meeting the requirements of factor analysis. In addition, the KMO statistic value is 0.704, which is >0.7. Moreover, Bartlett's sphericity test shows that the hypothesis of independence of each variable is not true (*P* = 0.000), indicating that factor analysis method should be used to weight the indicators.

#### Principal Component Analysis

The existing studies mainly used principal component analysis and entropy weighting method to measure each dimensional index, and then synthesized a comprehensive index of digital economic development. The entropy weighting method is an assignment method to objectively determine the weights based on the magnitude of variation of the indexes. However, in the evaluation index system, there may be a correlation among the indicators. Therefore, the traditional entropy weighting method has the problem of duplication of assignments, which leads to biased evaluation results. The principal component analysis method is able to screen out the main independent composite factors from many variables, which retains the original information while making them uncorrelated with each other. It is currently widely used in the construction of composite indicators for ICT and digital economy (12).

First, factor analysis is performed on the 11 indicators using the maximum variance rotation method to obtain three principal components with eigenvalues >1 and reflecting more than 70% of the data information (the cumulative contribution of variance is 70.08%). Secondly, the factor loading matrix is calculated using the principal component method. The initial unrotated factor loading matrix *A*_*i*_ is shown in Table 2.

**Table 2:** Initial unrotated factor loading matrix.

**Indicators**	**Principal component**		
	**1**	**2**	**3**
X1	0.550	−0.668	0.129
X2	0.447	0.002	0.138
X3	0.553	0.589	0.294
X4	0.527	0.684	0.142
X5	0.739	0.162	−0.444
X6	0.765	0.128	−0.315
X7	0.827	−0.398	−0.067
X8	0.650	−0.466	−0.261
X9	0.790	−0.242	0.207
X10	0.446	−0.114	0.725
X11	0.491	0.687	−0.127

*Data sources: SPSS22.0 software calculations*.

This study uses SPSS 22.0 software to establish the principal component rotated loading matrix Ui, which has a mathematical relationship with the factor loading matrix *A*_*i*_ and the eigenvalue λ_*i*_:


$$\begin{array}{l} U_{i}=\frac{A_{i}}{\sqrt{\lambda_{i}}} \end{array}$$

λ_*i*_ represents the eigenvalue corresponding to the i-th principal factor, reflecting the contribution of this principal factor to the total variance. The formula of *Y*_1_, *Y*_2_ and *Y*_3_ for the three principal components are obtained by multiplying *U*_*i*_ with the standard value (*Zx*_*i*_) of the 11 variables:


$$\begin{array}{l} Y_{1}=0.263Zx_{1}+0.213Zx_{2}+0.264Zx_{3}+0.252Zx_{4}+0.353Zx_{5}\\ \;+0.365Zx_{6}+0.395Zx_{7}+0.310Zx_{8}+0.377Zx_{9}+0.213Zx_{10}\\ \;+0.234Zx_{11}\\ Y_{2}=-0.448Zx_{1}+0.001Zx_{2}+0.395Zx_{3}+0.459Zx_{4}+0.109Zx_{5}\\ \;+0.086Zx_{6}-0.267Zx_{7}-0.313Zx_{8}-0.162Zx_{9}-0.076Zx_{10}\\ \;+0.461Zx_{11}\\ Y_{3}=0.123Zx_{1}+0.132Zx_{2}+0.281Zx_{3}+0.136Zx_{4}-0.424Zx_{5}\\ \;-0.301Zx_{6}-0.064Zx_{7}-0.249Zx_{8}+0.198Zx_{9}+0.692Zx_{10}\\ \;+0.121Zx_{11} \end{array}$$

Finally, the proportion of the corresponding eigenvalues to the three principal components to the total eigenvalues is taken as the weight to calculate the comprehensive scores, which is shown as follows:


$$\begin{array}{l} Y=\frac{\lambda_{1}}{\lambda_{1}+\lambda_{2}+\lambda_{3}}Y_{1}+\frac{\lambda_{2}}{\lambda_{1}+\lambda_{2}+\lambda_{3}}Y_{2}+\frac{\lambda_{3}}{\lambda_{1}+\lambda_{2}+\lambda_{3}}Y_{3} \end{array}$$

In this study, the comprehensive scores of each country in 2009–2019 are standardized and converted into values in the 0–100 interval. The standardization formula is presented as follows:


$$\begin{array}{l} \text{Score after standardization}=\left(X_{i}-X_{\min}\right)/\left(X_{\max}-X_{\min}\right)\times 100 \end{array}$$

In the above formula, *X*_i_ represents the original comprehensive score of country i; *X*_max_ and *X*_min_ represent the maximum and minimum scores of all countries, respectively. The calculation results are shown in Table 3.

**Table 3:** Comprehensive scores of digital economic development indicators of countries along the “Belt and Road” from 2009 to 2019.

**Ranking**	**Country**	**2009**	**2010**	**2011**	**2012**	**2013**	**2014**	**2015**	**2016**	**2017**	**2018**	**2019**
1	Singapore	75	77	74	74	76	76	80	84	90	90	100
2	Israel	61	59	61	61	60	61	68	69	71	71	74
3	Malaysia	59	60	60	62	62	66	68	67	67	67	71
4	Estonia	42	47	51	51	51	53	54	55	57	57	66
5	The Czech Republic	40	42	43	43	43	46	46	49	52	52	62
6	China	35	38	40	41	45	45	46	50	53	53	58
7	Vietnam	18	20	22	28	33	36	40	44	48	48	52
8	Hungary	44	46	44	40	39	38	36	42	45	45	50
9	Lithuania	34	34	35	37	37	38	40	41	43	43	49
10	Thailand	31	31	28	31	33	34	38	43	46	46	49
11	Slovenia	37	37	34	34	34	33	37	38	42	42	48
12	Latvia	27	27	30	34	37	40	43	41	40	40	45
13	Cyprus	46	47	42	36	35	36	34	34	39	39	41
14	Bulgaria	23	23	25	27	27	26	28	31	36	36	39
15	Greece	28	27	29	28	27	30	32	33	34	34	38
16	Saudi Arabia	28	33	37	35	34	32	35	34	34	34	38
17	Poland	29	29	28	29	29	32	33	34	36	36	38
18	Russian federation	23	25	22	25	28	31	33	33	33	33	37
19	Croatia	25	27	27	28	29	28	28	30	29	29	34
20	Romania	24	23	22	20	20	24	24	25	26	26	32
21	Kazakhstan	20	23	26	33	38	38	38	32	29	29	32
22	Oman	20	24	27	28	29	28	26	21	28	28	31
23	Azerbaijan	12	15	16	21	26	25	24	24	29	29	30
24	Armenia	1	9	10	14	15	16	18	21	21	21	25
25	Ukraine	15	14	16	20	19	20	22	20	21	21	25
26	Mongolia	6	6	9	10	10	12	7	12	10	10	22
27	India	14	13	15	14	14	11	13	17	18	18	21
28	The Republic of Egypt in Arabia	10	10	11	12	12	9	11	11	15	15	20
29	Moldova	3	7	10	11	13	16	16	15	18	18	20
30	Kyrgyzstan	2	0	3	4	5	8	13	15	16	16	14
31	Pakistan	1	3	4	4	4	2	3	3	8	8	13

*The ranking is based on the comprehensive score of 2019 digital economy development indicators for each country. Data sources: SPSS22.0 software calculations*.

### Analysis of Digital Economy Indicators

As shown in Table 3, the development of digital economy in countries along the “Belt and Road” showed an upward trend from 2009 to 2019. Most of the top 10 countries in the list are located in East Asia, Southeast Asia and Central and Eastern Europe, but not in South Asia and Central Asia. This suggests that there is an obvious regional imbalance in the digital economy development in countries along the “Belt and Road.” Although the comprehensive score of China's digital economy ranked sixth in the list, the score increased rapidly in 2009–2019. This indicates that in recent years, China has paid more attention to the digital economy, deepening the popularization and application of information technology. Therefore, the international competitiveness of digital economy industry has been gradually improved. According to the degree of digital economy development, the countries along the “Belt and Road” can be divided into the following three categories:

(1) Singapore, Israel and Malaysia were ranked in the top three countries along the “Belt and Road” in terms of comprehensive scores of digital economic development, with scores above 70 in 2019. Among them, Singapore ranked first, primarily because of its high scores on the three main components: the availability of the latest technology, the penetration of fixed broadband and the proportion of Internet users. This shows that Singapore has well-established information infrastructure, advanced information and communication technologies and high popularity and openness of digital economy.

(2) Estonia, Czech Republic, China, Vietnam, etc. had comprehensive scores of 30 to 70 in 2019. This indicates that the development of digital economy in these countries needs to be further strengthened. These countries had good performance in the fixed telephone penetration and mobile phone penetration, but other indicators were weak. Therefore, these countries still need to improve the digital economy infrastructure, and introduce favorable digital infrastructure policies to promote scientific and technological innovation, providing strong guarantees for the realization of high-quality development of digital economy.

(3) Armenia, Ukraine, Mongolia, India, Egypt, Moldova, Kyrgyzstan, and Pakistan were relatively backward in digital economy, with comprehensive scores <30 in 2019. Most of these countries are located in West Asia, Central Asia and South Asia. The infrastructure, professional talents, ICT capabilities and digital technology innovation environment required for the development of digital economy are seriously lacking in these countries. This indicates that there is a serious “digital divide” among the countries along the “Belt and Road.” Therefore, the countries that are lagging behind in the digital economy need to strengthen cooperation with others along the “Belt and Road” to make up for the shortcomings in the infrastructure and technological innovation capabilities.

## Digital Economy's Impact Before COVID-19

Based on the comprehensive scores of digital economy measured in Section Measurement of digital economy development, we use a panel data regression model and a mediating effects model to empirically test whether the digital economy played a significant role in promoting the economic growth of countries along the “Belt and Road” in the decade before COVID-19, and if so, what is the mechanism of this positive effect. This will help to further clarify the importance of digital economy development in the economic growth of countries along the “Belt and Road” and its impact path. It can also provide historical experience and evidence for taking the digital economy as an important stabilizer and booster for effective coordination of pandemic control and economic development in the post-COVID-19 era, thus promoting high-quality economic growth in countries along the “Belt and Road.”

### Data Sources and Statistical Description

This study uses the panel data of 31 countries along the “Belt and Road” from 2009 to 2019. The meaning and statistical description of the variables are shown in Table 4, including the dependent variable, the core independent variable, the mediating variables and the control variables. All variables are obtained from the World Bank database, except for the digital economy development scores, which are calculated from the previous section.

**Table 4:** Meaning of variables and statistical description.

	**Variable**	**Meaning**	**Mean**	**Std. Dev**.	**Min**	**Max**
The dependent variable	lngdppc	Logarithm of GDP per capita (2010 constant price dollars)	9.03	1.04	6.78	10.99
The core independent variable	digeco	Digital economy development scores	32.64	17.98	0.00	100.00
The mediating variables	lnservadd	Logarithm of services value-added as a share of total value-added (%)	3.98	0.18	3.32	4.33
	lnunemploy	Logarithm of total unemployment as a percentage of the total labor force (%)	1.77	0.75	−1.56	3.31
	lnservlabor	Logarithm of the share of service employment in total employment (%)	4.02	0.26	3.27	4.44
The control variables	lncapital	Logarithm of the gross fixed capital as a share of GDP (%)	3.11	0.27	2.32	3.88
	inflation	Annual inflation rate as measured by the consumer price index (%)	3.93	4.74	−2.10	48.70
	lnopen	Logarithm of total imports and exports as a share of GDP (%)	4.33	0.55	3.18	5.63
	FDI	Net foreign direct investment inflows as a share of GDP (%)	8.34	28.54	−40.33	280.13
	lngovern	Logarithm of general government final consumption expenditure as a share of GDP (%)	2.74	0.31	1.75	3.40

(1) The dependent variable. In this study, the logarithm of GDP per capita of the countries along the “Belt and Road” is taken as the dependent variable. The maximum value of the logarithm of GDP per capita of the countries along the “Belt and Road” is 10.99, the minimum value is 6.78, and the standard deviation is 1.04, indicating that the economic development level of these countries varies greatly. Among them, GDP per capita of Singapore, Israel, Cyprus, Slovenia, and the Czech Republic is higher than other countries, while that of India, Vietnam, Pakistan and Kyrgyzstan is below the mean value.

(2) The core independent variable. In this study, the digital economy development scores of countries along the “Belt and Road” are taken as the core independent variable. The average digital economy development score of these countries is 32.64; the maximum value is 100; the minimum value is 0; the standard deviation is 17.98, indicating that the digital economy development level of countries along the “Belt and Road” also varies greatly.

(3) Mediating variables. In order to investigate the impact of digital economy on the economic growth of countries along the “Belt and Road” through the effect of industrial structure, the total employment and employment structure, the proportion of value-added of service industry to the total value-added, unemployment rate and the proportion of service industry employment to the total employment are selected as mediating variables in this study.

(4) Control variables. Drawing on Habibi and Zabardast (43) and Myovella et al. (44), the following control variables are selected in this study: gross fixed capital formation as a share of GDP, annual inflation rate as measured by the consumer price index, total imports and exports as a share of GDP, net foreign direct investment inflows as a share of GDP, and government consumption expenditure as a share of GDP. These control variables simultaneously affect GDP per capita of countries along the “Belt and Road,” and must be controlled in the regression model to mitigate the bias caused by omitted variables.

### Model Setting

The impact of digital economy development on GDP per capita of countries along the “Belt and Road” is empirically examined by establishing a panel regression model:


(1) $$\begin{array}{l} \text{lngdpp}{\text{c}}_{\text{it}}={\text{α}}_{0}+{\text{α}}_{1}\text{digec}{\text{o}}_{\text{it}}+{\text{α}}_{2}\text{lncapita}{\text{l}}_{\text{it}}+{\text{α}}_{3}\text{inflatio}{\text{n}}_{\text{it}}\\ \;+{\text{α}}_{4}\text{lnope}{\text{n}}_{\text{it}}+{\text{α}}_{5}\text{FD}{\text{I}}_{\text{it}}+{\text{α}}_{6}\text{lngover}{\text{n}}_{\text{it}}+{\text{u}}_{\text{i}}\\ \;+{{\text{v}}_{\text{t}}+\text{ε}}_{\text{it}} \end{array}$$

where *gdppc*_*it*_ is GDP per capita of country i in period t, included in the regression equation in logarithmic form; *digeco*_*it*_ is the digital economy development score of country i in period t; *capital*_*it*_ is the gross fixed capital as a share of GDP of country i in period t; *inflation*_*it*_ is the annual inflation rate measured by the consumer price index of country i in period t; *open*_*it*_ is the trade openness of country i in period t, measured by the total imports and exports as a share of GDP; *FDI*_*it*_ is the share of net FDI inflows in GDP of country i in period t; *govern*_*it*_ is the share of government consumption expenditure in GDP of country i in period t; *u*_*i*_ is an individual fixed effect; *v*_*t*_ is a time fixed effect; ε_*it*_ is a random error term.

It is necessary to choose the appropriate regression model when processing panel data. Three forms of panel data regression models are usually chosen: pooled regression model (Pool), fixed effects regression model (FE), and random effects regression model (RE). The first step is to validate the model using the pooled regression model and use the F-test to determine whether the estimation method of pooled regression is used. The regression results show that none of the regression coefficients are significant, and the F-test results of the model reject the original hypothesis of using the pooled regression model at the 1% significance level, indicating that a variable intercept regression model should be built considering individual time characteristics. Further, the Hausman test is used to determine whether a fixed-effects model or a random-effects model is used for optimal estimation. The *p*-value rejects the original hypothesis that the random disturbance term is not related to the independent variables at the 1% significance level (*p* = 0.0000), indicating that the estimation results of establishing a fixed-effects model are optimal and most robust.

It is worth noting that the use of ordinary linear least square (OLS) estimation may have endogeneity problems, resulting in biased coefficient estimates of digital economy scores. The two-stage least squares (2SLS) and generalized method of moments (GMM) are designed to solve endogenous problems caused by omitted variables and reverse causality effects (45, 46). Some scholars suggested a reverse causal relationship between ICT and economic growth (44), and thus 2SLS and GMM techniques have been generally applied to assess the relationship between ICT and economic development (47, 48).

This study also investigates the impact mechanism of the development of digital economy on the economic growth of countries along the “Belt and Road” by selecting three mediating variables: the share of value-added of service industry in the total value-added, the unemployment rate and the share of employment in the service industry in the total employment. Among them, equations (2) and (3) are the mediating effects of the industrial structure; equations (4) and (5) are the mediating effects of the total employment; equations (6) and (7) are the mediating effects of the employment structure.


(2) $$\begin{array}{l} \text{lnservad}{\text{d}}_{\text{it}}={\text{β}}_{0}+{\text{β}}_{1}\text{digec}{\text{o}}_{\text{it}}+{\text{β}}_{2}\text{lncapita}{\text{l}}_{\text{it}}+{\text{β}}_{3}\text{inflatio}{\text{n}}_{\text{it}}\\ \;+{\text{β}}_{4}\text{lnope}{\text{n}}_{\text{it}}+{\text{β}}_{5}\text{FD}{\text{I}}_{\text{it}}+{\text{β}}_{6}\text{lngover}{\text{n}}_{\text{it}}+{\text{u}}_{\text{i}}\\ \;+{{\text{v}}_{\text{t}}+\text{ε}}_{\text{it}} \end{array}$$

(3) $$\begin{array}{l} \text{lngdpp}{\text{c}}_{\text{it}}={\text{γ}}_{0}+{\text{γ}}_{1}\text{digec}{\text{o}}_{\text{it}}+{\text{γ}}_{2}\text{lncapita}{\text{l}}_{\text{it}}+{\text{γ}}_{3}\text{inflatio}{\text{n}}_{\text{it}}\\ \;+{\text{γ}}_{4}\text{lnope}{\text{n}}_{\text{it}}+{\text{γ}}_{5}\text{FD}{\text{I}}_{\text{it}}+{\text{γ}}_{6}\text{lngover}{\text{n}}_{\text{it}}\\ \;+{\text{γ}}_{7}\text{lnservad}{\text{d}}_{\text{it}}{+\text{u}}_{\text{i}}+{{\text{v}}_{\text{t}}+\text{ε}}_{\text{it}} \end{array}$$

(4) $$\begin{array}{l} \text{lnunemplo}{\text{y}}_{\text{it}}={\text{β}}_{0}+{\text{β}}_{1}\text{digec}{\text{o}}_{\text{it}}+{\text{β}}_{2}\text{lncapita}{\text{l}}_{\text{it}}+{\text{β}}_{3}\text{inflatio}{\text{n}}_{\text{it}}\\ \;+{\text{β}}_{4}\text{lnope}{\text{n}}_{\text{it}}+{\text{β}}_{5}\text{FD}{\text{I}}_{\text{it}}+{\text{β}}_{6}\text{lngover}{\text{n}}_{\text{it}}+{\text{u}}_{\text{i}}\\ \;+{{\text{v}}_{\text{t}}+\text{ε}}_{\text{it}} \end{array}$$

(5) $$\begin{array}{l} \text{lngdpp}{\text{c}}_{\text{it}}={\text{γ}}_{0}+{\text{γ}}_{1}\text{digec}{\text{o}}_{\text{it}}+{\text{γ}}_{2}\text{lncapita}{\text{l}}_{\text{it}}+{\text{γ}}_{3}\text{inflatio}{\text{n}}_{\text{it}}\\ \;+{\text{γ}}_{4}\text{lnope}{\text{n}}_{\text{it}}+{\text{γ}}_{5}\text{FD}{\text{I}}_{\text{it}}+{\text{γ}}_{6}\text{lngover}{\text{n}}_{\text{it}}\\ \;+{\text{γ}}_{7}\text{lnunemplo}{\text{y}}_{\text{it}}{+\text{u}}_{\text{i}}+{{\text{v}}_{\text{t}}+\text{ε}}_{\text{it}} \end{array}$$

(6) $$\begin{array}{l} \text{lnservlabo}{\text{r}}_{\text{it}}={\text{β}}_{0}+{\text{β}}_{1}\text{digec}{\text{o}}_{\text{it}}+{\text{β}}_{2}\text{lncapita}{\text{l}}_{\text{it}}+{\text{β}}_{3}\text{inflatio}{\text{n}}_{\text{it}}\\ \;+{\text{β}}_{4}\text{lnope}{\text{n}}_{\text{it}}+{\text{β}}_{5}\text{FD}{\text{I}}_{\text{it}}+{\text{β}}_{6}\text{lngover}{\text{n}}_{\text{it}}+{\text{u}}_{\text{i}}\\ \;+{{\text{v}}_{\text{t}}+\text{ε}}_{\text{it}} \end{array}$$

(7) $$\begin{array}{l} \text{lngdpp}{\text{c}}_{\text{it}}={\text{γ}}_{0}+{\text{γ}}_{1}\text{digec}{\text{o}}_{\text{it}}+{\text{γ}}_{2}\text{lncapita}{\text{l}}_{\text{it}}+{\text{γ}}_{3}\text{inflatio}{\text{n}}_{\text{it}}\\ \;+{\text{γ}}_{4}\text{lnope}{\text{n}}_{\text{it}}+{\text{γ}}_{5}\text{FD}{\text{I}}_{\text{it}}+{\text{γ}}_{6}\text{lngover}{\text{n}}_{\text{it}}\\ \;+{\text{γ}}_{7}\text{lnservlabo}{\text{r}}_{\text{it}}{+\text{u}}_{\text{i}}+{{\text{v}}_{\text{t}}+\text{ε}}_{\text{it}} \end{array}$$

### Empirical Results and Analysis

The results show that the digital economy as an independent variable significantly contributes to the growth of GDP per capita in countries along the “Belt and Road” regardless of the inclusion of control variables (Table 5). Columns (1) and (2) report the OLS estimation results. The results in column (1) show that the regression coefficient of the digital economy is positive at 1% significance level without the inclusion of control variables, indicating that every 1-unit increase in the level of digital economy development will increase the GDP of the sample countries by 0.78%. This indicates that the development of new technologies related to the digital economy, such as the Internet and mobile communication, has a significant contribution to the economic growth of the countries along the “Belt and Road” from 2009 to 2019. The results in column (2) show that the regression coefficient of the digital economy is 0.00791 at the 1% significance level after controlling for variables such as the gross fixed capital, annual inflation rate, the total imports and exports as a share of GDP, net inflows of FDI, and the government final consumption expenditure, which means that each unit increase in the development level of the digital economy will increase the GDP of the sample countries by 0.79%. This indicates that the digital economy development has a significant contribution to the economic growth of the countries along the “Belt and Road.” The estimated results of 2SLS and dynamic differential GMM are shown in columns (3) and (4). The sign of the coefficients of the core independent variables does not change and the coefficients do not change significantly. For every 1 unit increase in the level of digital economy development, the GDP of the sample countries grows by 0.36–1.51%, indicating that the estimation results are still robust. For the GMM model, the Hansen test (Prob>chi2 = 0.627) and Arellano-Bond test for AR(2) (Pr>z = 0.444) indicate that the instruments are valid and there is no second-order autocorrelation in the difference of the random perturbation term.

**Table 5:** Impact of digital economy on GDP per capita of countries along the “Belt and Road.”

**Variables**	**(1)**	**(2)**	**(3)**	**(4)**
L1.lngdppc				0.838***
				(0.0458)
Digeco	0.00783***	0.00791***	0.0151***	0.00362***
	(0.00200)	(0.00111)	(0.00160)	(0.000786)
lncapital		0.0551*	−0.0292	0.0329
		(0.0310)	(0.0652)	(0.0278)
Inflation		−0.00191*	−0.00266**	−0.00101
		(0.00110)	(0.00114)	(0.00111)
lnopen		−0.0899**	−0.101	0.0308
		(0.0350)	(0.0719)	(0.0271)
FDI		−0.00002	−0.000452***	−0.0000801
		(0.000222)	(0.000172)	(0.000104)
lngovern		−0.166***	−0.176*	−0.0777***
		(0.0550)	(0.0951)	(0.0282)
Constant	8.691***	9.362***	9.566***	
	(0.0507)	(0.274)	(0.508)	
R-squared	0.650	0.672	0.5806	
AR(2)				0.444
Hansen test			0.627	

*Robust standard errors in parentheses, AR(2) and Hansen test report the p-value.*

***p < 0.01, **p < 0.05, *p < 0.1.

*L1.lngdppc is the first-order lag term of lngdppc*.

Our finding is consistent with the results of most studies. For example, it was found that the contribution of the ICT-based digital economy to GDP growth mostly ranged from 0.1 to 1.0 percentage points and tended to increase after 1995 (49). Obviously, our results are in the same direction but different in magnitude from other studies, which may be due to regional heterogeneity. Although digitalization can play a great role in economic growth, its impact may depend on the level of development of a country (50). Myovella et al. (44), for example, studied the relationship between digitization and economic growth in Sub-Saharan Africa and OECD using the GMM estimation method. They found that the contribution of the Internet to economic growth in both Sub-Saharan Africa and OECD was positive. However, due to the underdeveloped Internet infrastructure, the impact on Sub-Saharan Africa is smaller compared to OECD countries. This paper is focused on the countries along the “Belt and Road,” where the infrastructure is not well-established. However, with the promotion of the “Digital Silk Road,” job opportunities have been increased and the economic structure has been optimized, thus promoting the economic development of the countries.

It is worth noting that the regression coefficients of lnopen and FDI are negative. This indicates that the increase in import and export trade and net foreign direct investment inflows as a share of GDP in the countries along the “Belt and Road” does not contribute to GDP per capita. The increase of trade openness suppressed economic growth because the degree of openness in most countries did not match the level of economic development. According to descriptive statistics, the average level of trade openness of countries along the “Belt and Road” is low. In the early stage of opening up, most of the countries export labor-intensive and resource-intensive products to generate foreign exchange due to the lack of capital. This reliance on the low-end value chain may lead to a surge in exports in the short term, but it will lead to impoverished growth, which in the long run will have a dampening effect on economic development. In the future, as openness increases to a certain extent, the export structure will change, and domestic industrial upgrading will improve the quality of economic development and promote a more balanced development of trade, thus promoting the economic growth of countries along the “Belt and Road.” In addition, the share of net foreign direct investment inflows in GDP of some countries along the “Belt and Road” showed a decreasing trend from 2009 to 2019, indicating that the contribution of FDI to the economy of these countries decreased. This is mainly due to the low level of actual utilization of foreign investment in the countries along the “Belt and Road,” which leads to the failure of simultaneous economic growth. Although the increase in economic growth rate brought by FDI has a positive effect on the welfare of the invested countries, FDI may still reduce the welfare if the profits are transferred to foreign investors. Foreign investment increases welfare only when productivity gains are sufficient to compensate for the loss of profits. Our findings are also consistent with studies such as Li and Liu (51), Herzer and Klasen (52), and Ali and Abdullah (53).

Then, this study investigates the impact mechanism of the digital economy on the economic growth of countries along the “Belt and Road” by building mediating effects models. In Table 6, columns (1) and (2) report the results of the industrial structure effect mechanism. Column (1) shows that the digital economy can significantly promote the optimization and upgrading of industrial structure. The results show that 1-unit increase in the development level of the digital economy increases the value-added share of the service industry by 0.31%. Column (2) reports the impact of the digital economy on GDP per capita after adding the value-added share of the service industry to the base model. We find that the digital economy development can significantly increase the value-added share of the service industry, thus boosting the economic growth of countries along the “Belt and Road.” This is primarily because unlike the traditional agricultural and industrial production modes, the digital economy relies on the integration of modern information technology and network technology. This impact shows industrial heterogeneity, with the degree of impact gradually increasing from primary to tertiary industries. The difference in the rate of output increase in the digitalization process of different industries will bring about changes in the industrial structure. Therefore, the strong application ability of the tertiary industry to the digital economy can effectively promote the upgrading of industrial structure. Columns (3) and (4) report the results of mediating effect of total employment, while columns (5) and (6) report the results of employment structure effect. The results show that for every 1-unit increase in the level of development of the digital economy, the total unemployment rate significantly decreases by 1.15% and the share of employment in the service sector significantly increases by 0.11%. The digital economy can also contribute to the growth of GDP per capita in the countries along the “Belt and Road” by reducing the unemployment rate and improving the employment structure. This is primarily because the use of the Internet is becoming an important channel for job creation in most developing countries along the “Belt and Road.” The integration of ICT industries with traditional industries can lead to the expansion of economic scale, especially the growth of online consumption, and thus the effect of consumption-oriented jobs is gradually expanding. In addition, digital technology changes can also bring about restructuring of employment. Specifically, the development of digital economy can lead to the creation of more non-farm jobs, providing more employment opportunities and even increasing labor returns, which also increases the share of employment in the service industry.

**Table 6:** Test of mediating effects.

**Variables**	**(1)**	**(2)**	**(3)**	**(4)**	**(5)**	**(6)**
	**lnservadd**	**lngdppc**	**lnunemploy**	**lngdppc**	**lnservlabor**	**lngdppc**
Digeco	0.00312***	0.014***	−0.0115**	0.00736***	0.00114**	0.00736***
	(0.000936)	(0.000837)	(0.00453)	(0.0011)	(0.000555)	(0.00187)
lncapital	0.0115	0.00888	−0.683***	0.0226	−0.0682***	0.0883
	(0.0487)	(0.0366)	(0.126)	(0.032)	(0.0155)	(0.0733)
Inflation	−0.00113	−0.00275**	−0.0118***	−0.00247**	−0.000314	−0.00175
	(0.00107)	(0.00118)	(0.00448)	(0.00109)	(0.00055)	(0.00132)
lnopen	−0.106**	−0.0554	−0.21	−0.0999***	−0.0408**	−0.07
	(0.0431)	(0.0369)	(0.142)	(0.0345)	(0.0175)	(0.0645)
FDI	−0.000383***	−0.000276	−0.00143	−0.00008	−0.0001	0.00003
	(0.000118)	(0.000239)	(0.000903)	(0.000219)	(0.000111)	(0.000222)
lngovern	0.347***	−0.311***	0.28	−0.152***	0.00855	−0.17
	(0.116)	(0.0666)	(0.224)	(0.0542)	(0.0275)	(0.137)
lnservadd		0.23***				
		(0.0844)				
lnunemploy				−0.0476***		
				(0.0141)		
lnservlabor						0.487*
						(0.271)
Constant	3.355***	8.74***	4.503***	9.577***	4.311***	7.262***
	(0.466)	(0.413)	(1.117)	(0.277)	(0.137)	(1.178)
R-squared	0.37	0.581	0.267	0.684	0.533	0.691

*Robust standard errors in parentheses.*

***p < 0.01, **p < 0.05, *p < 0.1.

## COVID-19's Impact on Digital Economy

From the regression results in Section Digital economy's impact before COVID-19, it can be concluded that before COVID-19, the digital economy had a significant positive impact on the economic growth of countries along the “Belt and Road” by promoting industrial structure upgrading, the total employment and restructuring of employment. Although the outbreak of COVID-19 shocked the economies along the “Belt and Road” from both the supply and demand sides (54, 55), home isolation measures promoted the development of new businesses such as e-commerce, telemedicine, and online offices, increasing demand for the digital economy (56). Therefore, we use the GTAP model to analyze the impact of COVID-19 on the output and trade patterns of digital industries of countries along the “Belt and Road,” and to identify the regional heterogeneity of the potential of digital industries during the epidemic. This will help to seize the opportunities for digital economy to mitigate the economic losses, and propose differentiated development strategies to realize economic recovery and growth in the post-COVID-19 era.

It is worth noting that the two major forms of the digital economy are digital industrialization and industrial digitization. Digital industrialization means the development of ICT industries, including electronic information manufacturing, telecommunications, software and information technology services, and the Internet industry (57). Industrial digitization deeply integrates advanced digital technologies with traditional industries. This accelerates the transformation and upgrading of traditional industries, and improves their production efficiency (58). Considering the applicability of the model and data availability, this study focused on the impact of the COVID-19 epidemic on the digital industry, including the sector of computers, communication and other electronic equipment manufacturing, and information transmission services.

### Methods and Simulation Scenarios

Due to the short duration of the epidemic and the lack of empirical data, it is hard to use econometric models based on ex-post analysis. Therefore, we use the GTAP model which is based on ex-ante analysis. The model transmits external shocks through a global multi-regional MRIO table, which is widely used in policy simulations. For example, Zeshan (59) uses the GTAP-VA model to simulate the impact of COVID-19 on global value chains and evaluate the production losses in different sectors of the world economy.

#### Methods

The GTAP model is a general equilibrium model developed by Purdue University and applied to global trade analysis. The GTAP model takes the production, consumption and government expenditure as sub-models, and connects the sub-models into a multi-country, multi-sector general equilibrium model through the relationship of commodity trade between countries or regions. When simulations are performed in the GTAP model, the data are based on inter-international input-output tables and external shocks are transmitted through the input-output tables. Moreover, the GTAP model can simultaneously evaluate the impact of policies on indicators such as output, imports and exports, and gross domestic product in each country sector, and is therefore widely used in policy analysis.

This study used the GTAP model and the most recent GTAP database: Version 10, including 65 industries and 141 countries or regions worldwide. In view of the completeness and availability of the shock variable, the 141 countries or regions in the GTAP 10 database were divided into 24 countries along the “Belt and Road” (including China, Malaysia, Indonesia, and the Philippines etc.), other countries along the “Belt and Road,” developed countries (including the United States, the United Kingdom, Japan, South Korea, and 16 countries in the European Union other than countries along the “Belt and Road”), and other countries or regions. The specific country and regional classifications are shown in Appendix Table 2. In addition, this study divided the 65 industries in GTAP 10 into 5 sectors, namely, agriculture, energy, manufacturing, service, and digital industry. The specific industry classifications are shown in Appendix Table 3.

#### Shock Variables and Simulation Scenarios

The COVID-19 epidemic affected the economic system of each region mainly from the supply side and demand side in this study. The supply-side shock variable was labor; the demand-side shock variables were consumption, investment, and preference for digital industries such as computers and communications. The specific impact paths are as follows.

First, the COVID-19 epidemic affected the labor supply. The rising number of people infected or killed by COVID-19 led to a reduction in labor supply. In addition, the epidemic prevention measures taken by some countries to restrict the movement of people (60), as well as the business decisions made by firms to lay off workers (61), reduced labor force participation rates (62), leading to a temporary shortage of labor supply and a decrease in the country's total output.

Second, the COVID-19 epidemic caused a decrease in total consumption. During the outbreak, enterprises in some countries shut down their production due to the lock-down policies and the isolation measures, causing an increase in unemployment and a decrease in individual income (63). This issue led to lower household consumption and increased precautionary savings (64, 65). It was also made it more difficult for residents to consume due to travel restrictions.

Third, the COVID-19 epidemic led to a decline in investment. Faced with the downward pressure on economic performance and the increased uncertainty in the financial market (66), investors chose their investments more cautiously (67). As a result, the demand for investment decreased.

Fourth, the COVID-19 epidemic spawned the needs of the digital industry. Although the total demand declined during the epidemic, with the vigorousness of big data-based epidemic prevention, distance learning, artificial intelligence, computer, communication and other electronic equipment manufacturing, and information transmission services showed greater potential during the COVID-19 (68). Therefore, the epidemic instead increase people's preference for digital industries.

Corresponding to the above analysis, this study set up the shock variables according to the impact mechanism of COVID-19 epidemic. The magnitude of the impact of various factors on the economy, that is, the value of the shock variables, was determined by the rate of change of each variable in 2020 compared to 2019. The data of the shock variables were obtained from World Development Indicators (WDI) and International Monetary Fund (IMF). This study used the rate of change of labor force to measure the degree of change of labor force relative to the base period; the rate of change of final consumption expenditure to measure the degree of change of consumption relative to the base period; the rate of change of gross fixed capital formation to measure the degree of change of investment relative to the base period; and the rate of change of the share of consumption of computers, communications and other services to measure the change of people's preference for digital industries. The detailed description and value of each shock variable in each region are shown in Table 7.

**Table 7:** Values of supply-side and demand-side shocks in the simulated scenarios.

**Region**	**Supply-side**	**Demand-side**		
	**Labor force**	**Consumption**	**Investment**	**Preference for digital industries**
CHN	−1.66%	−0.91%	3.24%	8.34%
MYS	0.78%	−2.87%	−15.79%	6.63%
IDN	−0.87%	−2.10%	−7.22%	13.40%
PHL	−3.11%	−5.24%	−24.83%	9.73%
IND	−4.66%	−7.11%	−15.14%	3.49%
PAK	−2.06%	−2.01%	−6.61%	5.09%
POL	−0.62%	−1.56%	−7.85%	4.48%
CZE	−1.16%	−2.61%	−7.01%	2.23%
SVK	−0.97%	−0.68%	−9.62%	4.93%
HUN	−0.38%	−1.95%	−4.43%	3.04%
SVN	−0.53%	−6.75%	−1.15%	6.51%
HRV	−0.71%	−4.12%	−2.17%	7.92%
ALB	−1.37%	−2.00%	0.02%	8.72%
EST	−0.68%	−0.64%	17.74%	18.23%
LTU	−0.32%	−1.45%	3.55%	4.63%
LVA	−0.13%	−6.95%	3.48%	7.71%
UKR	−3.69%	0.48%	−25.16%	5.40%
BLR	−1.91%	−1.44%	−14.23%	3.20%
TUR	−3.01%	3.02%	−0.58%	−0.22%
ISR	−0.57%	−5.87%	−0.13%	13.75%
ARM	−5.23%	−10.20%	−2.74%	7.24%
GEO	0.01%	5.42%	−7.26%	7.47%
EGY	−2.35%	7.25%	−8.91%	−2.89%
OBLT	−1.28%	−1.87%	−6.91%	5.08%
DEV	−1.01%	−2.33%	−1.67%	6.65%
ROW	−3.59%	−4.28%	−10.61%	8.76%

*Data sources: World Development Indicators (WDI) and International Monetary Fund (IMF)*.

According to the characteristics of the COVID-19 epidemic affecting macro-economy and industries, this study set up three different scenarios in the policy simulation, denoted by S1, S2, and S3 respectively. The shock was first applied to the labor force on the supply side (S1), then to the preferences of consumption, investment, and digital industries on the demand side (S2). Finally, a superimposed shock was applied to both the supply and demand sides (S3).

### Simulation Results and Discussion

**Figure 1:**
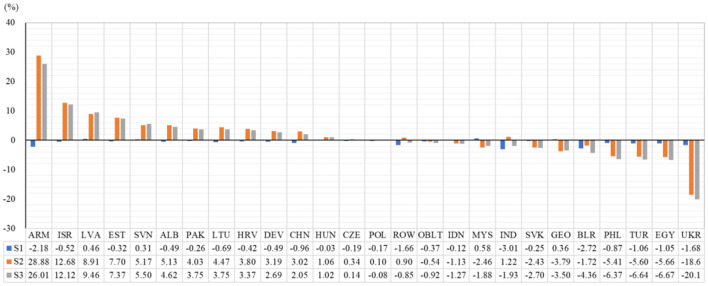
Changes in the output of the digital industries under the three simulation scenarios (unit: %). Data sources: GTAP model simulations.

Figure 1 shows the output changes of the digital industries under three simulation scenarios. Overall, the impact of the COVID-19 epidemic on the digital industries is primarily determined by the demand side, that is, the impact of the shock from demand side on the digital industries is far larger than that from the supply side.

From the perspective of the supply side, in the simulation of the labor shock (S1), the output of digital industries in almost all regions declined to various degrees, but the magnitude of the changes was small. This is consistent with the reality. When the COVID-19 epidemic occurred, in order to reduce the risk of gathering and infection, most countries and regions in the world adopted blockade measures to significantly reduce the frequency of people's travel (69). Labor is an important factor of production for economic activities. Therefore, labor shortage will lead to a greater impact on the output of productive sectors, especially labor-intensive sectors (70). However, the digital industries are capital-intensive and high-tech, which have much lower labor demand than the manufacturing and energy industries. And teleworking during the COVID-19 epidemic can also help the digital industries to resume work and production, mitigating the impact on the digital industries due to labor shortage. Our simulation results also confirm this conclusion. Appendix Table 4 details the changes in the output of all regions and industries in the S1 simulation. As shown in column 5 of Appendix Table 4, the changes in output for the digital industries across all regions in the S1 simulation fluctuated between −3.01 and 0.58%. The average rate of change for the digital industries across all regions is much lower than the energy and manufacturing industries.

Comparing the results of demand-side shocks (S2) and superimposed shocks on the supply side and demand side (S3), we find that the impact of the two scenarios on the output of digital industries showed the same trend in most regions. This suggests that relative to a labor shortage crisis, shocks to consumption, investment, and preference have a greater impact on the output of the digital industries.

**Figure 2:**
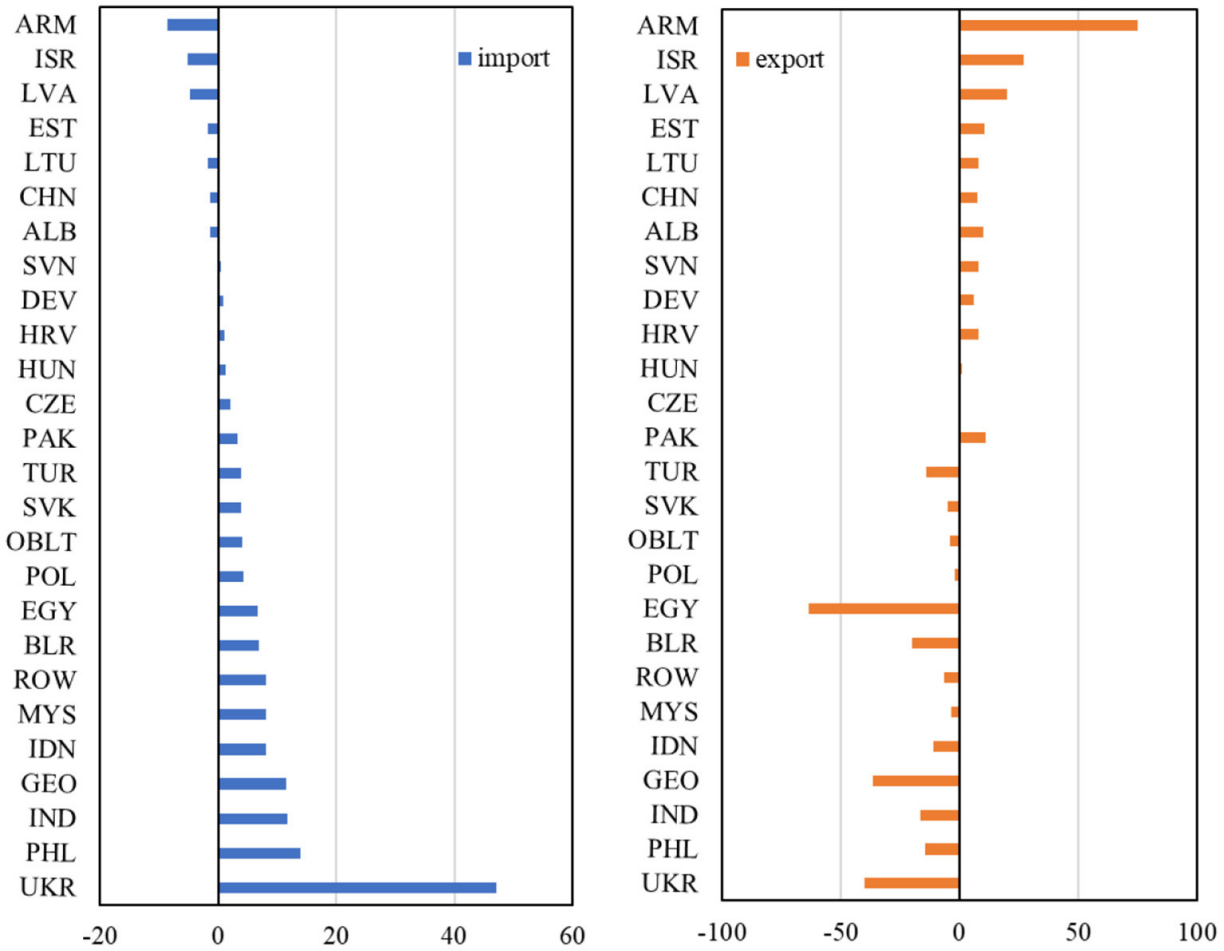
Changes in digital industry exports and imports by region in S3 simulation (unit: %). Data sources: GTAP model simulations.

Specifically, the digital industries in Armenia (ARM) located in the Middle East, Israel (ISR) in West Asia, and Latvia (LVA) and Estonia (EST) in Central and Eastern Europe showed large growth during the COVID-19 epidemic. Under the superimposed supply-side and demand-side shocks (S3), the output of the digital industries in the four countries increased by 26.01%, 12.12%, 9.46%, and 7.37% respectively. Figure 2 illustrates the changes in the exports and imports of each country under the S3 simulation. The rapid growth of the output of digital industries in Armenia, Israel, Latvia, and Estonia were all attributed to a decrease in imports and an increase in exports. Under the impact of the COVID-19 epidemic, imports decreased by 8.61%, 5.18%, 4.75%, and 1.74%, respectively; exports increased by 74.93%, 26.83%, 20.01%, and 10.64%, respectively. This shows that the products of these countries' digital industries are becoming more competitive. In addition, the products produced can not only meet the increase in domestic demand, but also can realize import substitution and export expansion, together promoting the increase of the output of digital industries.

The reason is that Israel and Estonia have a solid foundation in the digital industries. It can be seen from Table 3, Israel and Estonia are ranked 2nd and 4th, respectively. This indicates that the digital industries of the two countries have a high level of development among the “Belt and Road” countries. Latvia performs well in digital public services, due to the continuous improvement of local network infrastructure and the growing popularity of e-government services (71). Armenia also has a series of unique advantages in the digital industries, such as strong R&D capabilities in computer science and engineering, a highly educated workforce, strong government support for the digital industries, and the extensive operational management experience of large multinational companies (72). Based on the strong digital industry foundation, the COVID-19 epidemic led to an increase in people's preference for digital products. Therefore, the epidemic became a “booster,” which promoted the development of the digital industries (73).

It was worth noting that China's digital industries also stood out in the COVID-19 epidemic. Although the output of China's digital industries had a relatively small rate of change compared with regions such as Armenia, it had a large base. Therefore, the rapid development of China's digital industries is important for promoting the “Digital Silk Road” and the “Innovation Silk Road.” As can be seen in Figure 1, in the S3 simulation, COVID-19 epidemic did not hinder the development of China's digital industries, but increased the output by 2.05%. In addition, the net exports of China's digital industries also increased during the COVID-19 epidemic. As shown in Figure 2, the imports decreased by 1.26% and exports increased by 7.52%. As an emerging economy that fully utilizes computers, and information and communication technologies (74), China's digital industries have gradually become important supporting force for economic development. Therefore, during the COVID-19 epidemic, the government supervision and community management, monitoring and prevention in healthcare, as well as the life services that residents need for home study, work and life, have generated demand and output growth for the digital industries in China (75–77).

However, there were also some countries where the digital industries performed poorly during the COVID-19 epidemic, such as Ukraine (UKR), Egypt (EGY), Turkey (TUR), and Philippines (PHL). Under the superimposed shocks on the supply side and the demand side (scenario 3), their output of the digital industries decreased by 20.14%, 6.67%, 6.64%, and 6.37%, respectively. In addition, it can be found that the imports increased and exports decreased due to the impact of the COVID-19 epidemic in Ukraine, Egypt, Turkey and the Philippines (see Figure 2). Their imports increased by 47.13%, 6.70%, 3.85%, and 13.92%, respectively; exports decreased by 39.97%, 63.53%, 13.93%, and 14.64%, respectively. It was also noteworthy that Ukraine and the Philippines showed an increased preference for digital industry under the impact of the COVID-19 epidemic. Therefore, they had to increase imports and reduce exports to fill the increased domestic demand for digital products.

The negative impact of COVID-19 on Ukraine's output of digital industries is primarily due to the low coverage of information technologies among the residents and enterprises. The labor migration, technological backwardness and gradual loss of competitive position on the international market, make it difficult for the development of its digital industries to seize the opportunities. Similarly, the Philippines' digital industry infrastructure is also relatively backward, and its digital industry exports are gradually shrinking in the global and Association of Southeast Asian Nations (ASEAN) region. Additionally, Turkey's digital industries have low contributions to the economy because of its high investment costs, unstable rate of returns, and lack of skilled labor when using information technology to achieve industrial change. These have become obstacles in the path of the development of the digital industries in Turkey.

## Conclusions and Policy Implications

This study constructed a comprehensive evaluation index system and used principal component analysis to measure the digital economy development level of countries along the “Belt and Road” from 2009 to 2019. Then, a panel data regression model was applied to empirically analyze the impact of digital economy on their economic growth before COVID-19. Finally, we used the GTAP model to examine the impact of COVID-19 on the digital economy and its trade pattern of countries along the “Belt and Road.” Our findings show that: (1) there exists an obvious regional imbalance in the development of digital economy in countries along the “Belt and Road.” Specifically, East Asia, Southeast Asia (especially for Singapore), and Central and Eastern Europe have relatively high levels of digital economy, while most countries in West Asia (except for Israel), Central Asia, and South Asia are still lagging behind. (2) The digital economy has a significantly positive effect on the economic growth in countries along the “Belt and Road.” It can stimulate economic growth by promoting industrial structure upgrading, the total employment and restructuring of employment. (3) COVID-19 has generally boosted the demand for digital industries in countries along the “Belt and Road,” and its impact on digital industries from the demand side is much larger than that from the supply side. Specifically, the digital industries in Armenia, Israel, Latvia and Estonia have shown great growth potential during the epidemic. However, COVID-19 has also brought negative impacts to the digital industries in Ukraine, Egypt, Turkey and the Philippines. Accordingly, this study proposes the following policy implications:

(1) Each country along the “Belt and Road” should identify its strengths and weaknesses based on the digital economy development scores, so as to formulate effective development strategies and paths. The network infrastructure in Central Asia and South Asia is backward, so it is important to alleviate and bridge the “digital divide” and assist the countries or regions with less-developed digital technologies. Due to the large development gap within regions, the more backward countries can learn from countries such as Singapore, Israel, Malaysia, and explore the appropriate development models in the light of their own status. These countries should focus on strengthening R&D support for frontier digital technologies such as artificial intelligence and 5G, enhancing specialized talent training, and improving the innovation environment for digital economy.

(2) In the post-COVID-19 era, attention needs to be paid to the driving effect of the digital economy on industrial upgrading and employment. On the one hand, economic globalization and information technology need to be combined to further promote the deep integration of the digital economy with traditional primary, secondary and tertiary industries. Countries along the “Belt and Road” need to enhance the digital management and operation of traditional industries through ICT technology, optimize the efficiency of industrial resources allocation, improve their economic efficiency and increase the value-added of industries. On the other hand, the important role of the digital economy as a stabilizer for the job market needs to be utilized well. In the post-COVID-19 era, there will be a significant increase in the demand for digital living, working and learning. This is a rare opportunity for the development of the digital economy. Therefore, countries along the “Belt and Road” should use the employment promotion mechanism of the digital economy to promote digital employment, thus improving labor efficiency and contributing to steady economic recovery and growth.

(3) In the post-COVID-19 era, countries along the “Belt and Road” should strengthen the cooperation in the digital economy, and promote the deep integration of the real economy and the digital economy, industrialization and informatization. They should improve the digital connectivity, promote the information technology, and create new growth points for cooperation through building the “Digital Silk Road.” During the COVID-19 pandemic, the demand for digital life, work and learning in countries along the “Belt and Road” has increased significantly, which is a rare opportunity for the development of the digital economy. Therefore, those countries should further improve the development strategies of digital economy, optimize and upgrade the construction of information infrastructures such as artificial intelligence, internet of things and industrial internet. In addition, a favorable environment for the development of digital firms should be created to support them to increase investment in digital technologies.

(4) Countries along the “Belt and Road” need to rely on the digital economy to develop new service trade patterns and increase the cooperation in digital trade and e-commerce. COVID-19 has led to the disruption of both human and logistic flows, forcing the digital transformation of traditional trade in goods and services. On the contrast, digital trade will become the main form of global trade driven by emerging digital technologies such as big data, cloud computing, artificial intelligence and blockchain. Therefore, countries along the “Belt and Road” need to improve their technological innovation capabilities, and expand the range of cooperation in terms of service trade, such as cross-border e-commerce, teleconferences and exhibitions, telemedicine, tele-education and unlimited payment, so as to promote the construction of a “digital trade community.”

(5) As the initiator of the “Belt and Road” initiative, China should not only promote its own digital economy development, but also focus on the long-term layout for “Digital Silk Road.” China can take advantage of its technical advantages in the cooperation of digital economy in countries along the “Belt and Road” through economic exchanges and assistance. In addition, effective measures need to be taken to encourage Chinese information enterprises to invest and export abroad and to provide high-quality information technology products to countries along the “Belt and Road.” Furthermore, China needs to help those countries that lack the conditions to build their own improve their network infrastructure, in order to achieve the goal of the “Belt and Road” connectivity construction.

## Data Availability Statement

The original contributions presented in the study are included in the article/Supplementary Material, further inquiries can be directed to the corresponding author.

## Author Contributions

JZ designed the research framework, analyzed the data, and drafted the original manuscript. WZ performed calculation and analyzed the data. BC verified and solidified the argument, and edited the article. AL and YW participated in data collection. NY and YT revised the manuscript during the whole writing process. All authors contributed to the drafting of the article and read the final manuscript.

## Funding

This study was supported by National Science Fund for National Natural Science Foundation of China (71873016, 72073012), Beijing Social Science Foundation (21LLGLC038), Humanities and Social Science Foundation of Ministry of Education of China (21YJC630127), and Social Science Program of Beijing Municipal Education Commission (SM202011417010).

## Conflict of Interest

NY was employed by Beijing Shenzhou Chiji Fund Management Co., Ltd. The remaining authors declare that the research was conducted in the absence of any commercial or financial relationships that could be construed as a potential conflict of interest.

## Publisher's Note

All claims expressed in this article are solely those of the authors and do not necessarily represent those of their affiliated organizations, or those of the publisher, the editors and the reviewers. Any product that may be evaluated in this article, or claim that may be made by its manufacturer, is not guaranteed or endorsed by the publisher.
