# Supplementary material for: The Impact of Digital Economy on the Economic Growth and the Development Strategies in the post-COVID-19 Era: Evidence From Countries Along the “Belt and Road”
Source: Front Public Health. 2022 May 9;10:856142. doi: 10.3389/fpubh.2022.856142 (PMC9164196; doi:10.3389/fpubh.2022.856142)
Supplement: Supplementary file 1 [file Table_1.docx]

**Appendix**

**Appendix Table 1 The 7 major regions and corresponding countries along the “Belt and Road”**

| Region | Country |
| --- | --- |
| East Asia | China |
| Central Asia | Kazakhstan, Kyrgyzstan, Tajikistan, Turkmenistan, Uzbekistan |
| North Asia | Mongolia, Russia |
| Southeast Asia | Vietnam, Laos, Cambodia, Thailand, Malaysia, Singapore, Indonesia, Brunei, Philippines, Myanmar, East Timor |
| South Asia | India, Pakistan, Bangladesh, Afghanistan, Nepal, Bhutan, Sri Lanka, Maldives |
| Central and Eastern Europe | Poland, Czech Republic, Slovakia, Hungary, Slovenia, Croatia, Romania, Bulgaria, Serbia, Montenegro, Macedonia, Bosnia and Herzegovina, Albania, Estonia, Lithuania, Latvia, Ukraine, Belarus, Moldova |
| West Asia and the Middle East | Turkey, Iran, Syria, Iraq, UAE, Saudi Arabia, Qatar, Bahrain, Kuwait, Lebanon, Oman, Yemen, Jordan, Israel, Palestine, Armenia, Georgia, Azerbaijan, Egypt |

**Appendix Table 2 Country and Region Classifications in GTAP model**

| Country Classification | Countries or regions included |
| --- | --- |
| Countries along the “Belt and Road” | China, Malaysia, Indonesia, Philippines, India, Pakistan, Poland, Czech Republic, Slovak Republic , Hungary, Slovenia, Croatia, Albania, Estonia, Lithuania, Latvia, Ukraine, Belarus, Turkey, Israel, Armenia, Georgia, Egypt |
| Other countries along the “Belt and Road” | Kazakhstan, Kyrgyz Republic , Tajikistan, Turkmenistan, Uzbekistan, Mongolia, Russian Federation, Vietnam, Laos, Cambodia, Thailand, Singapore, Brunei, Myanmar , East Timor, Bangladesh, Afghanistan, Nepal, Bhutan, Sri Lanka, Maldives, Romania, Bulgaria, Serbia, Montenegro , North Macedonia, Bosnia and Herzegovina, Moldova, Iran, Syrian Arab Republic, Iraq, United Arab Emirates, Saudi Arabia, Qatar, Bahrain, Kuwait, Lebanon, Oman, Yemen, Jordan, Palestine, Azerbaijan |
| Developed countries | United States, United Kingdom, Japan, South Korea, Austria, Belgium, Cyprus, Denmark, Finland, France, Germany, Greece, Ireland, Italy, Luxembourg, Malta, Netherlands, Portugal, Spain, Sweden |
| Other countries or regions | Other 55 countries or regions except China, developed countries and countries along the “Belt and Road” |

**Appendix Table 3** **Industry Classification for GTAP Simulation Solutions**

| Industry Classification | Products |
| --- | --- |
| Agriculture | Paddy rice; Wheat; Cereal grains; Vegetables, fruit, nuts; Oil seeds; Sugar cane, sugar beet; Plant-based fibers; Crops; Bovine cattle, sheep and goats; Animal products; Raw milk; Wool, silk-worm cocoons; Forestry; Fishing; Bovine meat products; Meat products; Vegetable oils and fats; Dairy products; Processed rice; Sugar; Food products; Beverages and tobacco products |
| Energy | Coal; Oil; Gas; Minerals |
| Manufacturing | Textiles; Wearing apparel; Leather products; Wood products; Paper products, publishing; Petroleum, coal products; Chemical products; Basic pharmaceutical products; Rubber and plastic products; Mineral products; Ferrous metals; Metals; Metal products; Motor vehicles and parts; Transport equipment; Manufactures |
| Service | Electricity; Gas manufacture, distribution; Water; Construction; Trade; Accommodation, Food and service; Transport; Water transport; Air transport; Warehousing and support activity; Financial services; Insurance; Real estate activities; Business services; Recreational and other service; Public Administration and defence; Education; Human health and social work; Dwellings |
| Digital Industry | Computer, electronic and optical products; Electrical equipment; Machinery and equipment; Communication |

**Appendix Table 4 Changes in output of all regions and industries in S1 simulation (unit: %)**

|  | Agriculture | Energy | Manufacturing | Service | Digital Industries |
| --- | --- | --- | --- | --- | --- |
|  | (1) | (2) | (3) | (4) | (5) |
| CHN | -0.74 | -1.31 | -0.99 | -0.84 | -0.96 |
| MYS | 0.15 | -0.74 | 0.45 | 0.46 | 0.58 |
| IDN | -0.34 | -0.89 | -0.37 | -0.27 | -0.12 |
| PHL | -0.85 | -2.22 | -1.06 | -1.30 | -0.87 |
| IND | -1.74 | -1.21 | -2.40 | -2.62 | -3.01 |
| PAK | -0.57 | -0.81 | -0.71 | -0.57 | -0.26 |
| POL | -0.30 | -1.06 | -0.50 | -0.20 | -0.17 |
| CZE | -0.42 | -1.12 | -0.84 | -0.35 | -0.19 |
| SVK | -0.37 | -2.36 | -0.64 | -0.26 | -0.25 |
| HUN | -0.17 | -1.01 | -0.56 | -0.12 | -0.03 |
| SVN | -0.14 | -1.83 | -0.85 | -0.23 | 0.31 |
| HRV | -0.36 | -0.89 | -0.59 | -0.37 | -0.42 |
| ALB | -0.39 | -0.72 | -0.48 | -0.65 | -0.49 |
| EST | -0.31 | -0.68 | -0.83 | -0.28 | -0.32 |
| LTU | -0.53 | -1.95 | 0.06 | -0.01 | -0.69 |
| LVA | 0.02 | -3.05 | -0.72 | 0.03 | 0.46 |
| UKR | -1.21 | -1.30 | -1.26 | -2.68 | -1.68 |
| BLR | -1.29 | -2.25 | -1.30 | -0.78 | -2.72 |
| TUR | -0.96 | -1.93 | -1.24 | -1.02 | -1.06 |
| ISR | -0.38 | -2.54 | -0.29 | -0.38 | -0.52 |
| ARM | -2.50 | -3.62 | -3.47 | -2.40 | -2.18 |
| GEO | 0.06 | -1.90 | -0.52 | 0.00 | 0.36 |
| EGY | -0.88 | -0.58 | -1.07 | -1.31 | -1.05 |
| OBLT | -0.51 | -0.74 | 0.28 | -0.48 | -0.37 |
| DEV | -0.52 | -0.89 | -0.70 | -0.60 | -0.49 |
| ROW | -1.39 | -0.70 | -1.47 | -2.09 | -1.66 |
